# Neurovascular dysfunction, inflammation and endothelial activation: Implications for the pathogenesis of Alzheimer's disease

**DOI:** 10.1186/1742-2094-8-26

**Published:** 2011-03-25

**Authors:** Paula Grammas

**Affiliations:** 1 Garrison Institute on Aging, and Department of Neurology, Texas Tech University Health Sciences Center, Lubbock, Texas, USA

## Abstract

Alzheimer's disease (AD) is an age-related disorder characterized by progressive cognitive decline and dementia. Alzheimer's disease is an increasingly prevalent disease with 5.3 million people in the United States currently affected. This number is a 10 percent increase from previous estimates and is projected to sharply increase to 8 million by 2030; it is the sixth-leading cause of death. In the United States the direct and indirect costs of Alzheimer's and other dementias to Medicare, Medicaid and businesses amount to more than $172 billion each year. Despite intense research efforts, effective disease-modifying therapies for this devastating disease remain elusive. At present, the few agents that are FDA-approved for the treatment of AD have demonstrated only modest effects in modifying clinical symptoms for relatively short periods and none has shown a clear effect on disease progression. New therapeutic approaches are desperately needed. Although the idea that vascular defects are present in AD and may be important in disease pathogenesis was suggested over 25 years ago, little work has focused on an active role for cerebrovascular mechanisms in the pathogenesis of AD. Nevertheless, increasing literature supports a vascular-neuronal axis in AD as shared risk factors for both AD and atherosclerotic cardiovascular disease implicate vascular mechanisms in the development and/or progression of AD. Also, chronic inflammation is closely associated with cardiovascular disease, as well as a broad spectrum of neurodegenerative diseases of aging including AD. In this review we summarize data regarding, cardiovascular risk factors and vascular abnormalities, neuro- and vascular-inflammation, and brain endothelial dysfunction in AD. We conclude that the endothelial interface, a highly synthetic bioreactor that produces a large number of soluble factors, is functionally altered in AD and contributes to a noxious CNS milieu by releasing inflammatory and neurotoxic species.

## Introduction

Alzheimer's disease (AD) is an age-related disorder characterized by progressive cognitive decline and dementia. Alzheimer's disease is an increasingly prevalent disease with 5.3 million people in the United States currently affected; it is the sixth-leading cause of death. The direct and indirect costs of Alzheimer's and other dementias to Medicare, Medicaid and businesses amount to more than $172 billion each year [1]. Despite intense research efforts, effective disease-modifying therapies for this devastating disease remain elusive.

The clinical entity AD has, by definition, been categorized as a "non-vascular" dementia. Widely used diagnostic criteria classify dementia as either vascular or AD-driven; despite the reality of clinical practice where vascular comorbidity may be present in 30%-60% of AD patients and, conversely, AD pathology may be present in 40%-80% of vascular dementia patients [2]. Because of its classification as a non-vascular dementia, the role of neuro-vascular interactions in the evolution of neuronal injury in AD brain has been underappreciated. Nevertheless, increasing literature supports a vascular-neuronal axis in AD as shared risk factors for both AD and atherosclerotic cardiovascular disease implicate vascular mechanisms in the development and/or progression of AD.

### Cardiovascular risk factors in AD

Numerous studies link vascular risk factors to cognitive decline and dementia in the elderly [3-32]. Old age, atherosclerosis, stroke, hypertension, transient ischemic attacks, cardiac disease, the epsilon 4 allele of the apolipoprotein E (ApoE), elevated homocysteine levels, hyperlipidemia, metabolic syndrome, obesity and diabetes are risk factors for both vascular dementia and AD [5-7,10-16]. Homocysteine, considered an independent risk factor for vascular disease, has also been shown to increase the risk of AD [7,16]. Several studies have shown a high correlation between cardiovascular mortality and AD and an association among hypertension, diabetes and dementia [21,23-28]. Inheritance of the ApoE allele ε4 increases the risk of developing both atherosclerosis and late-onset AD, suggesting a vascular component to the pathogenesis of neuronal degeneration in AD [5]. There is increasing evidence identifying a link between heart disease and AD [2,8-15,17,19,20]. Heart disease is a prevalent finding in AD, and may be a forerunner to the dementing disorder. Also, increased prevalence of AD-like amyloid beta (Aβ) deposits in the neuropil and within neurons occurs in the brains of non-demented individuals with heart disease [3,4]. There is a three-fold increase in risk of developing AD or vascular dementia in people with severe atherosclerosis [6]. The large population-based Rotterdam study finds that atherosclerosis, primarily in the carotid arteries, is positively associated with the risk of developing dementia [18]. Postmortem grading of Circle of Willis atherosclerotic lesions shows that atherosclerosis is more severe in cases with AD and vascular dementia than in non-demented controls [22]. Finally, the idea that vascular dysfunction is a primary/central event in the pathogenesis of AD has been proposed in the context of a two-hit model of AD pathogenesis [19,32]. This hypothesis postulates that neurovascular damage is a primary occurrence and that subsequent injuries including Aβ deposition amplify and/or exacerbate vascular damage which then leads to neurodegenerative processes/events and ultimately cognitive decline.

### Functional and structural cerebrovascular abnormalities in AD

Abnormalities in the vascular system of the brain could contribute to the onset and/or progression of neurodegenerative events in AD [33]. Elevated levels of markers of endothelial dysfunction (E-selectin, vascular cell adhesion molecule 1(VCAM-1)) have been determined in the plasma of older subjects with late onset AD and vascular dementia [34]. Data from brain imaging studies in humans and animal models suggest that cerebrovascular dysfunction precedes cognitive decline and the onset of neurodegenerative changes in AD and AD animal models [35,36]. Emission tomography including single photon emission computed tomography (SPECT) and positron emission tomography (PET) show AD is characterized by bilateral temporoparietal hypoperfusion on SPECT and hypometabolism on PET which precede onset of dementia [37]. The Alzheimer's Disease Neuroimaging Initiative (ADNI) has examined longitudinal change in glucose metabolism using [(18)F]-fluorodeoxyglucose PET (FDG-PET) and finds the use of FDG-PET as an outcome measure in clinical trials increases statistical power over traditional cognitive measurements, aids in subject selection, and reduces clinical trial sample size [38].

The idea that vascular defects are present in AD and may be important in disease pathogenesis was suggested over 25 years ago [39]. The two-hit hypothesis of AD pathogenesis emphasizes the primary role of vascular defects and data linking brain endothelial cell products with neuronal cell death in AD, further support a central role for vascular abnormalities in disease pathogenesis [19,32,33]. Numerous structural and functional cerebromicrovascular abnormalities in AD have been identified [40-56]. These are atrophy and irregularities of arterioles and capillaries, swelling and increased number of pinocytotic vesicles in endothelial cells, increase in collagen IV, heparin sulfate proteolglycans and laminin deposition in the basement membrane, disruption of the basement membrane, reduced total microvascular density and occasional swelling of astrocytic end feet [39,41,42,45,48,56]. Ultrastructural analysis of the blood-brain barrier in AD patients demonstrates decreased mitochondrial content, increased pinocytosis, accumulation of collagen and focal necrotic changes [48,50,56]. Structural changes in cerebral capillaries in elderly patients correlate positively with advanced age and dementia [39]. Also, vascular distortions such as vessel kinking, twisting, tortuosity, and looping occur in AD [44]. Neuronal cell loss in AD may result from pathologic changes in vessel angioarchitecture, decreased cerebral blood flow, and altered oxygen utilization leading to cerebral microcirculatory impairment. Microvascular pathology displays regional and laminar patterns that parallel patterns of neuronal loss [45]. There is a topographic association of capillaries with neuritic plaques [40,43]. In addition, vascular-derived heparin sulfate proteolglycan deposits co-localize with senile plaques [45]. A study using vascular corrosion casts to visualize the 3D arrangement of the brain vasculature, shows that in young AD animals, lacking parenchymal amyloid plaques, significant morphologic abnormalities of blood vessels are evident [57]. Reduced staining of endothelial markers CD34 and CD31 observed in AD brains suggests that there is an extensive degeneration of the endothelium during the disease progression [47]. Taken together, these data suggest profound vascular perturbations in AD.

### Neuroinflammation, vascular inflammation and the pathogenesis of AD

Data suggest that there are important pathogenic mechanisms common to both Alzheimer's and cardiovascular disease. Chronic inflammation, characterized by elevated plasma concentrations of C reactive protein, a plasma acute-phase protein, is associated with an increased risk of atherosclerosis and has been documented in the lesions of AD [58-60]. Inflammation, by definition a vascularized tissue response to injury, is a key connector linking vascular abnormalities and AD pathogenesis. A wide range of inflammatory cytokines and chemokines has been documented to play a role in the evolution of the atherosclerotic plaque. Indeed, vascular inflammation, especially of the endothelium, is central to the initiation and progression of the atherosclerosis [61,62]. Chronic inflammation is associated with a broad spectrum of neurodegenerative diseases of aging including AD [63]. Numerous studies show the presence of markers of inflammation in the AD brain [64-71]. Elevated cytokines and chemokines as well as the accumulation of activated microglia are found in or near the pathologic lesions of AD [65,70]. In animal models of AD lipopolysaccharide (LPS)-induced inflammation has been shown to exacerbate phosphotau pathology [72].

Retrospective epidemiological studies suggest that a wide variety of non-steroidal anti-inflammatory drugs (NSAIDs) may significantly reduce one's lifetime risk of developing AD [73-76]. These drugs inhibit the enzymatic activity of cyclooxygenase-1 (COX-1) and inducible COX-2 which catalyze the first committed step in the synthesis of prostaglandins. Inducible COX-2 is elevated in AD; both COX-1 and 2 are involved in numerous inflammatory activities. COX inhibitors can decrease levels of highly amyloidogenic Aβ1-42 peptide [77]. Long-term use of NSAIDs is also associated with protection from the development of AD and reduction in Aβ deposition in mouse models of AD and is positively correlated with reduction of plaque-associated microglia in both humans and mice [78-82]. A meta-analysis of epidemiological data shows that NSAIDs reduce AD incidence by an average of 58% [83]. Despite considerable animal and retrospective human studies that show beneficial effects of anti-inflammatory drugs in mitigating AD pathology, NSAIDs have failed to demonstrate therapeutic benefit in prospective AD clinical trials [84-86]. No detectable effects on a variety of clinical outcome measures of AD progression are found in controlled trials of naproxen, celecoxib, and rofecoxib [84,86]. Results of a clinical trial with more than 2500 participants show no significant cognitive improvement after 4 years of treatment with either naproxen or celecoxib [86]. A large phase III trial of fluriproen recently provided no evidence of cognitive improvement in AD subjects [85].

Increasingly the timing of anti-inflammatory administration appears critical. The Cache county study suggests that NSAIDs help prevent cognitive decline in older adults if started in midlife rather than late life [87]. In a study in AD transgenic mice, the reentry of neurons into the cell cycle, a pathologic feature of AD, is prevented but not reversed by NSAIDs, suggesting that inflammation is important for initiation but not the progression of the disease process [88]. It is likely that the neurodegeneration observed in AD is the result of pathogenic processes including inflammation initiated well before the onset of cognitive symptoms associated with the disease. Recent findings suggest that alterations in production of inflammatory cytokines and chemokines are early features that precede Aβ deposition in mouse models of AD [88-90]. Early expression of inflammatory mediators in the AD brain by non-neuronal cells, including endothelial cells, is likely critical to the development of disease.

In AD there is a robust elevation in inflammatory mediators in the cerebral microcirculation. AD brain endothelial cells express high levels of inflammatory adhesion molecules such as monocyte chemoattractant protein-1 (MCP-1) intercellular adhesion molecule-1 (ICAM-1) and cationic antimicrobial protein 37 kDa (CAP37) [54,91,92]. Compared to microvessels from age-matched controls, AD brain microvessels release significantly higher levels of a number of inflammatory factors including nitric oxide (NO), thrombin, tumor necrosis factor-α (TNFα), transforming growth factor-β (TGF-β), interleukin (IL) IL-1β, IL-6, IL-8, and matrix metalloproteinases (MMPs) [46,54,55,93]. The cerebral microvasculature is a participant in a destructive cycle of events where inflammation precedes Aβ deposition and Aβ in turn promotes release of inflammatory mediators. In this regard, exposure of brain endothelial cells to Aβ has been shown to evoke an array of pro-inflammatory responses. Aβ, via its interaction with the receptor for advanced glycation end products (RAGE) up-regulates CCR5 expression and promotes T cell migration across the blood-brain barrier [94]. Monocytes are transported across the BBB via Aβ-RAGE-mediated signaling [95]. Inhibitors of c-Jun NH2-terminal kinase (JNK), extracellular signal-regulated protein kinase (ERK), and phosphatidylinositol 3'-kinase (PI3K) signaling cascades significantly decrease Aβ-induced CCR5 expression in human brain endothelial cells [94]. Cultured brain endothelial cells exposed to Aβ1-40 up-regulate expression of inflammatory genes MCP-1, IL-1β, and IL-6. Quantitative RT-PCR analysis confirms elevated expression of these genes in AD and AD/CAA brains [96]. Treatment of isolated brain microvessels with Aβ results in an increase in prostaglandin production [97]. Cultured brain endothelial cells exposed to Aβ express CD40 and secrete interferon-gamma and IL-1β [98]. The cerebromicrocirculation is a dynamic interface serving as both a source of, and a target for, inflammatory proteins.

### Inflammation and angiogenesis: implications for AD

The mechanisms whereby inflammatory events and mediators contribute to AD pathogenesis are unclear. Specifically, the link between vascular inflammation and neuronal dysfunction and death, pathognomonic abnormalities in the AD brain, has not been defined. In the periphery, chronic inflammation as a regulator of angiogenesis has been documented [99,100]. The finding that inflammation is often associated with increased angiogenesis is explained by inflammation-induced production of pro-angiogenic factors.

The possible linkage of inflammation and angiogenesis in the brain has not been widely examined in AD. However, data are emerging to support the idea that factors and processes characteristic of angiogenesis are found in the AD brain. Genome-wide expression profiling in the AD brain has identified a marked upregulation of genes that promote angiogenesis [101]. Cerebral hypoperfusion is one of the major clinical features in AD and likely plays a critical role in its pathogenesis [102]. Hypoxia is known to stimulate angiogenesis as well as contribute to the clinical and pathological manifestations of AD [102,103]. Hypoxia causes upregulation of hypoxia-inducible genes such as vascular endothelial growth factor (VEGF) [103,104]. VEGF, a potent mediator of angiogenesis, is present in the AD brain in the walls of intraparenchymal vessels, diffuse perivascular deposits, and in clusters of reactive astrocytes [105]. In addition, intrathecal levels of VEGF in AD are related to clinical severity and to intrathecal levels of Aβ [106]. Increasing evidence suggests that polymorphisms within the promoter region of the VEGF gene may elevate the risk for AD [107]. Brain microvessels express and release a large number of inflammatory proteins (1L-1β, IL-6, IL-8, TNFα, TGFβ, MCP-1); many of which have been implicated in angiogenesis [54,55,93] In addition, the vasculature demonstrates up-regulation of specific molecules thought to be important regulators/markers of the angiogenic process including thrombin, VEGF, angiopoietin-2, integrins (α_V_β_3 _α_V_β_5_), and hypoxia inducible factor 1α (HIF-1α) [93,108,109]. The notion that inflammation and angiogenesis are interrelated molecular events is supported by studies showing that IL-1β, a major proinflammatory cytokine, can substitute for key aspects of hypoxia signaling, including induction of VEGF gene expression [101,110]. This may be especially relevant for AD pathophysiology as high levels of this cytokine in AD- but not control-derived microvessels have been reported [54]. Taken together, these data suggest a heretofore-unexplored connection between angiogenesis and AD.

Despite increases in several pro-angiogenic factors in the AD brain, evidence for increased vascularity in AD is lacking. On the contrary, it has been suggested that the angiogenic process is delayed and/or impaired in aged tissues, with several studies showing decreased microvascular density in the AD brain [111-113]. Work from Mullan's group has shown that wild-type Aβ peptides have antiangiogenic effects *in vitro *and *in vivo *[114]. Also, impaired angiogenesis has been demonstrated in AD transgenic mice [115]. Recent data show that an amyloid peptide Aβ1-40 (E22Q), derived from individuals with the rare autosomal dominant disorder hereditary cerebral hemorrhage with amyloidosis-Dutch type, has stronger anti-angiogenic activity than wild-type Aβ peptides and is poorly cleared from the brain [116,117]. This appears to be related to the increased formation of low molecular weight Aβ oligomers in the culture medium surrounding human brain microvascular endothelial cells. The notion that amyloid may serve as a "brake'" in the angiogenic process is also supported by data which show that plaque-derived amyloid inhibits brain endothelial cell proliferation *in vitro *[118]. Recent genomic profiling of brain endothelial cells shows low levels of vascular-restricted mesenchyme homeobox 2 gene (MEOX2) in AD individuals lead to aberrant angiogenesis and premature pruning of capillary networks resulting in reductions in the cerebral microcirculation and that hypoxia suppresses MEOX2 expression in brain endothelial cells [119]. Furthermore, in that study a transgenic mouse line deficient in MEOX2 gene displays vascular regression and poor Aβ clearance.

**Figure 1:**
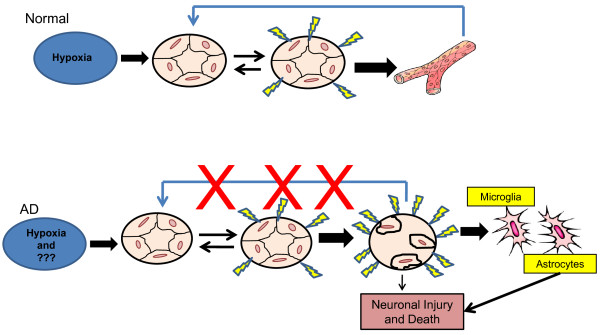
**Diagram of hypothesis**. In response to a persistent or intermittent stimulus, such as cerebral hypoxia, brain endothelial cells become activated. Activated endothelial cells are highly synthetic and release a host of factors that can affect the activation of nearby cells. Despite the continued presence of the stimulus, no new vessel growth occurs. Because no new vessels are formed there are no feedback signals to shut off vascular activation endothelial cells, as occurs in physiologic angiogenesis. In AD reversible endothelial activation becomes irreversible endothelial dysfunction. The vascular products of a permanently dysfunctional endothelium could cause neuronal injury/death directly or via activation of microglia and/or astrocytes. (blue line) = feedback inhibition, (yellow lighting bolt) = Endothelial cell products

The angiogenic process is complex and involves several discrete steps beginning with endothelial activation. The activated endothelium synthesizes and secretes a large number of pro-angiogenic mediators [120,121]. In physiologic angiogenesis endothelial activation is reversible and self-limiting. We propose a working model to reconcile the seemingly contradictory observations of both a large number of pro-angiogenic mediators and an absence of new vessel growth in the AD brain. In response to a persistent or intermittent stimulus, such as cerebral hypoperfusion, one of the major clinical features of AD, brain endothelial cells become activated. Activated endothelial cells are highly synthetic and release a host of factors that can affect the activation of nearby cells (i.e. astrocytes, microglia) and/or the viability of neurons. Despite the continued presence of the stimulus, no new vessel growth occurs. The inhibition of vascular growth is likely mutlifactorial and could involve the anti-angiogenic activity of Aβ, defective homeobox signaling, as well as combinations of these and other mechanisms [115-119]. Because no new vessels are formed, there are no feedback signals to shut off vascular activation endothelial cells. In AD reversible endothelial activation becomes irreversible endothelial dysfunction. The vascular products of a permanently dysfunctional endothelium could cause neuronal injury. A diagram of this hypothesis is shown in Figure 1.

Results of epidemiological studies suggest that some drugs purported to have beneficial effects in AD inhibit angiogenesis [122-125]. The idea that vascular inflammatory changes that promote angiogenesis have functional consequences in AD is supported by data suggesting that some of the beneficial effects of NSAIDs are linked to anti-angiogenic activity [126-128]. NSAIDs might inhibit angiogenesis through several mechanisms. These drugs can directly inhibit angiogenic signaling pathways such as mitogen-activated ERK2 in endothelial cells [129]. NSAIDs can also inhibit endothelial growth factor expression and subsequent migration [130]. Also, the enzymes COX-1 and COX-2, targets of NSAIDs, are important regulators of angiogenesis [131]. The efficacy of COX-2 selective inhibitors in AD treatment may be related to the inhibition of prostaglandins, NO and TNFα, all of which are important to both inflammation and angiogenesis [131]. Thalidomide, another anti-angiogenic drug which blocks endothelial cell activation and suppresses release of VEGF and TNFα, appears protective in animal models of AD preventing memory impairment induced by Aβ [132,133]. Also, statins, purported to have beneficial effects in AD, have anti-angiogenic effects [134-136].

### Endothelial cell activation and neuronal injury

Endothelial cells are key modulators of inflammation and angiogenesis [62]. The endothelium is a common target for all cardiovascular risk factors, and functional impairment of the vascular endothelium in response to injury occurs long before the development of overt disease [137-139]. Chronic inflammation is tightly linked to diseases associated with endothelial dysfunction. Phenotypic modulation of endothelium to a dysfunctional state is recognized to contribute to the pathogenesis of cardiovascular diseases such as atherosclerosis [140,141]. Endothelial dysfunction is also increasingly implicated in the development of neurodegenerative diseases such as AD [19,32,33,142-146]. Brain endothelial cells regulate the neuronal milieu both by their synthetic functions as well as by their blood-brain barrier function. Therefore, disturbance in cerebrovascular metabolic or transport functions could result in a noxious neuronal environment in the AD brain.

#### Synthetic functions of endothelial cells in health and disease

The endothelium is a highly synthetic interface that produces a large number of soluble factors; a partial list of these products is shown in Table 1[147]. Endothelial cell products have critical effects on neighboring cells. In the walls of large vessels endothelial cell products affect smooth muscle cell phenotype and contribute to the evolution of the atherosclerotic plaque [141]. Vascular-derived products of a permanently dysfunctional endothelium could result in neuronal injury in neurodegenerative disease states. In the AD brain, an injured/altered brain endothelial cell releases factors that are injurious or toxic to neurons [33]. Evidence for vascular-mediated neuronal cell death in AD is derived from studies where direct co-culture of AD microvessels with neurons or incubation of cultured neurons with conditioned medium from microvessels results in neuronal cell death [52]. In contrast, vessels from elderly nondemented donors are significantly less lethal and brain vessels from younger donors are not neurotoxic. A study using cultured brain endothelial cells shows that exposure of these cells to the inflammatory proteins IL-1β and LPS causes release factors that kill cholinergic neurons [148]. Also, inflammatory or oxidant injury of brain endothelial cells *in vitro *leads to release of the neurotoxic protease thrombin [149]. Finally, the importance of the neurovascular unit as a mediator of neuronal damage is highlighted by a recent study where pericyte-deficient mice show an age-dependent vascular damage that precedes neurodegenerative changes and cognitive impairment [150].

**Table 1:** Secretory/Expression Products of Endothelial Cells*

*Extracellular matrix factors*	*Proteases*
fibronectin	Thrombin

laminin	MMPs

collagen I, II, III, IV, VIII, XVIII	tPA

proteoglycans	***Growth factors***

***Anti- and pro- coagulation factors***	PDGF

PGI_2_	EDGF

thrombomodulin	FGF

AT III	IGF

heparin sulfate	TGF-β

vWF	GM-CSF

TXA_2_	G-CSF

thromboplastin	***Vasorelaxation factors***

Factor V	NO

PAF	PGI_2_/E_2_

PAI-1, PAI-2	EDHF

***Inflammatory Chemokines and Cytokines***	***Vasoconstriction factors***

IL-1, IL-6, IL-8	TXA_2_/F_2a_

LTB_4_, C_4_, D_4_, E_4_	EDCF

MCP-1, MCP-2	leukotrienes

MHC II	free radicals

CAM	endothelin

*See text for references

Abbreviations used: prostacyclin (PGI_2_), anti-thrombin III (AT III), von Willebrand factor (vWF), thromboxane A2 (TXA_2_), platelet-activating factor (PAF), plasminogen activator inhibitor-1 (PAI-1), plasminogen activator inhibitor-2 (PAI-2), interleukin-1 (IL-1), interleukin-6 (IL-6), interleukin-8 (IL-8), leukotriene B4 (LTB_4_), leukotriene C4 (LTC_4_), leukotriene D4 (LTD_4_), leukotriene E4 (LTE_4_), monocyte chemoattractant protein-1 (MCP-1), monocyte chemoattractant protein-2 (MCP-2), major histocompatibility complex class II (MHC II), cell adhesion molecules (CAM), matrix metalloproteinases (MMPs), tissue plasminogen activator (tPA), platelet-derived growth factor (PDGF), endothelium derived growth factor (EDGF), fibroblast growth factor (FGF), insulin-like growth factor (IGF), transforming growth factor beta (TGF-β), granulocyte macrophage colony-stimulating factor (GM-CSF), granulocyte colony-stimulating factor (G-CSF), nitric oxide (NO), prostacyclin/prostaglandin E2 (PGI_2_/E_2_), endothelium derived hyperpolarizing factor (EDHF), thromboxane A2/prostaglandin F2a (TXA_2_/F_2a_), endothelium derived contracting factor (EDCF)

In AD thrombin has been detected in the senile plaques, characteristic of this disease [151]. Traumatic brain injury where neurons are exposed to high thrombin levels is associated with an increased incidence of AD [152,153]. Some neurologic diseases, such as AD and PD are characterized by increased levels of both thrombin and the thrombin receptor protease-activated receptor 1 (PAR-1) [154,155]. Furthermore, immunoreactivity for the major brain thrombin inhibitor, protease nexin-1 is found to be significantly decreased in AD brains, particularly around blood vessels, suggesting vascular release of thrombin [156]. Our laboratory has shown, by RT-PCR, that brain blood vessels isolated from AD patients, but not age-matched controls, synthesize thrombin [109]. Thrombin is an example of a vascular-derived factor relevant for AD because of its pluripotent effects on inflammation, angiogenesis and neurotoxicity.

Thrombin causes endothelial activation and enhanced expression and/or release of many proinflammatory proteins including MCP-1 and ICAM-1, both of which are upregulated in the cerebrovasculature in AD [54,91,157]. The cellular action of thrombin, potent angiogenic factor, on endothelial cells may represent an important early event in activation of the normally quiescent endothelial cells and initiation of the angiogenic cascade. In endothelial cells, thrombin induces their alignment in Matrigel, the expression and secretion of angiopoietin-2, MMPs, IL-1β, IL-8 and the up-regulation of VEGF receptors [158,159]. In addition, thrombin stimulates upregulation of integrin αVβ3 expression in endothelial cells [160]. These results are likely to be important for understanding vascular pathology in AD as vascular expression of VEGF, angiopoietin-2, MMPs, IL-1β, IL-8, and integrins are all upregulated in the AD brain [54,93,108].

The multifunctional protease thrombin causes neuronal cell death both *in vitro *and *in vivo *[161-169]. Thrombin causes rapid tau aggregation [165]. Intracerebroventricular administration of thrombin directly into the rat brain results in neuronal cell death, glial scarring and cognitive deficits [166]. Activation or over-expression of the receptor PAR-1 has been shown to induce motor neurodegeneration [162]. Thrombin exerts direct neurotoxicity by several mechanisms including reentry into the cell cycle, induction of pro-apoptotic proteins, as well as via NADPH oxidase mediated oxidative stress [169,170].

Furthermore, the paracrine effects of thrombin released from endothelial cells are also important because of the ability of thrombin to activate other CNS cells such as microglia and astrocytes. Pro-inflammatory effects of thrombin on both microglia and astrocytes have been demonstrated. Intranigral injection of thrombin injures the dopaminergic neurons in the substantia nigra via thrombin-induced microglial activation and release of nitric oxide [171]. Thrombin has been shown to stimulate the JAK2-STAT3 signaling pathway and increase transcription of inflammation-associated genes TNFα and inducible nitric oxide synthase in microglia [172]. In astrocytes, activation of PAR-1 by thrombin leads to increased MMP-9 expression through regulation of ERK1/2 [173]. Thus, vascular-derived thrombin may directly injure neurons or affect neuronal viability indirectly via activation of microglia and astrocytes in the neurovascular unit.

#### Neurovascular unit dysfunction and amyloid transport

The neurovascular unit is an emerging concept that emphasizes the interactions among glial, neuronal and vascular elements [17,174-177]. Homeostatic signaling within the neurovascular unit is critical to normal brain function. The hemodynamic communication between neurons and the cerebrovasculature is necessary to efficiently couple CBF to neuronal activation. Dysfunctional cell-cell signaling in the neurovascular unit is increasingly implicated as characteristic feature of CNS diseases [19,175,177-179]. Structural and functional integrity of the CNS depends on the coordinated activity of the neurovascular unit to not only couple neural activity to CBF but also to regulate transport across the blood-brain barrier. There is some evidence that disturbance of the functional relationships among the cells of the neurovascular unit is an early event in AD [17]. Functional MRI studies suggest that alterations in CBF regulation in response to cognitive tasks may be a predictor of risk for developing AD [37].

An important function of the blood-brain barrier that may go awry in AD is regulation of the brain pool of Aβ. Brain Aβ, which is in equilibrium with plasma and CSF Aβ, is modulated by influx of soluble Aβ across the blood-brain barrier via its interaction with the receptor for advanced glycation end products (RAGE) and efflux via the low density lipoprotein receptor on brain endothelial cells. Accumulating evidence from patients and animal models of AD suggests that vulnerable brains may suffer from an increase in influx receptors (RAGE) and/or a decrease in efflux receptors (lipoprotein receptor-related protein) [180,181]. Multiple pathogenic cascades in the neurovascular unit may contribute to faulty clearance of amyloid across the blood-brain barrier which may amplify neuronal dysfunction and injury in AD.

Aβ has toxic effects on endothelial cells both via direct mechanisms and by inducing local inflammation [94,96-98]. The cerebral microvasculature is central to a destructive cycle of events where inflammation precedes Aβ deposition and Aβ in turn promotes release of inflammatory mediators. The cerebrovascular-derived inflammatory protein thrombin, via stimulation of functionally active thrombin (PAR-1 and PAR-3) receptors [182] on brain endothelial cells can further stimulate inflammatory processes in an autocrine fashion. Thrombin *in vitro *can stimulate production of the amyloid precursor protein (APP) and cleavage of APP into fragments that are found in amyloid plaques of AD brains [183,184]. In this manner, Aβ and thrombin may combine to stimulate a deleterious feed-forward cycle resulting in neuronal cell death in AD.

## Conclusions

Despite intense research efforts, the enigma that is AD continues to present daunting challenges for effective therapeutic intervention. The lack of disease-modifying therapies may, in part, be attributable to the narrow research focus employed to understand this complex disease. Most human and animal studies in the AD field reflect a "neurocentric" view and have focused on the Aβ protein as the primary neurotoxic species involved in disease pathogenesis. Because of its classification as a non-vascular dementia, the role of neuro-vascular interactions in the evolution of neuronal injury in AD brain has been underappreciated. Nevertheless, increasing literature supports a vascular-neuronal axis in AD as shared risk factors for both AD and atherosclerotic cardiovascular disease implicate vascular mechanisms in the development and/or progression of AD. The endothelium is a common target for all cardiovascular risk factors, and functional impairment of the vascular endothelium in response to injury occurs long before the development of overt disease. Chronic inflammation, a feature of AD, is tightly linked to diseases associated with endothelial dysfunction. The cerebromicrocirculation is a dynamic interface serving as both a source of, and a target for, inflammatory proteins. Brain endothelial cells regulate the neuronal milieu both by their synthetic functions as well as by their blood-brain barrier function. Therefore, disturbance in cerebrovascular metabolic or transport functions could result in a noxious neuronal environment in the AD brain. The cerebral microvasculature is central to a destructive cycle of events where inflammation precedes Aβ deposition and Aβ in turn promotes release of inflammatory mediators. In this review we summarize data that support a new paradigm of disease pathogenesis, based on endothelial dysfunction and release of pluripotent mediators with effects on inflammation, vascular activation/angiogenesis and neurotoxicity. The activated/dysfunction brain endothelium is a novel, unexplored therapeutic target in AD.

## Competing interests

The author declares that they have no competing interests.

## Authors' contributions

PG wrote the manuscript and has approved the final version of the manuscript.
